# Plant plasma membrane proteomics for improving cold tolerance

**DOI:** 10.3389/fpls.2013.00090

**Published:** 2013-04-17

**Authors:** Daisuke Takahashi, Bin Li, Takato Nakayama, Yukio Kawamura, Matsuo Uemura

**Affiliations:** ^1^United Graduate School of Agricultural Sciences, Iwate UniversityMorioka, Iwate, Japan; ^2^Cryobiofrontier Research Center, Iwate UniversityMorioka, Iwate, Japan

**Keywords:** plasma membrane, cold acclimation, freezing tolerance, abiotic stress, shotgun proteomics, gel-based proteomics

## Abstract

Plants are always exposed to various stresses. We have focused on freezing stress, which causes serious problems for agricultural management. When plants suffer freeze-induced damage, the plasma membrane is thought to be the primary site of injury because of its central role in regulation of various cellular processes. Cold tolerant species, however, adapt to such freezing conditions by modifying cellular components and functions (cold acclimation). One of the most important adaptation mechanisms to freezing is alteration of plasma membrane compositions and functions. Advanced proteomic technologies have succeeded in identification of many candidates that may play roles in adaptation of the plasma membrane to freezing stress. Proteomics results suggest that adaptations of plasma membrane functions to low temperature are associated with alterations of protein compositions during cold acclimation. Some of proteins identified by proteomic approaches have been verified their functional roles in freezing tolerance mechanisms further. Thus, accumulation of proteomic results in the plasma membrane is of importance for application to molecular breeding efforts to increase cold tolerance in crops.

## INTRODUCTION

Survival of plants is considerably dependent on their ability to adapt to environmental conditions under which they live. Because of their immobility, plants need to recognize and respond to external stimuli sensitively and properly for their survival. Without proper signal perception and subsequent responses, they can be killed or injured rapidly and instantaneously. External stimuli to influence plant’s life include a number of different factors resulting from biotic stresses (e.g., microorganism infection and wounding by herbivores) and abiotic stresses (e.g., drought, salt, flooding, light, nutrition, physical pressure, and extreme temperatures).

The plasma membrane, one of the semi-permeable cellular membranes, is the membrane that separates the intracellular and extracellular spaces, and plays an important role in the exchange of compounds such as metal ions, metabolites, and nutrients. Signals from extracellular stimuli may also be transmitted through the plasma membrane, most of which are likely conducted by plasma membrane proteins. In addition, the plasma membrane itself needs to withstand stresses from the extracellular space. Therefore, plant cells that perceive stress stimuli are thought to alter the proteomic properties of the plasma membrane to adapt to stresses. Management of the effects of these stress-related stimuli is important for improvement of growth performance, yield and quality of crops in the field. For example, crops growing in most American farmlands potentially face threats from drought, heat, and cold stresses, and it was estimated that drought and heat stresses caused damage of US$4.2 billion in August 2000 (Mittler, 2006).

Many plants that live in temperate regions, including important crop species such as wheat, rye, and barley, increase their freezing tolerance when air temperature decreases and day length shortens, which is called cold acclimation (Levitt, 1980). In particular, alterations of plasma membrane proteins during cold acclimation have been well characterized using biochemical and physiological approaches and recognized as a critical adaptation mechanism to low temperature (Steponkus, 1984; Uemura and Yoshida, 1984; Uemura et al., 2006). More specifically, during cold acclimation, increased P-type ATPase activity, disassembly of microtubules and accumulation of several dehydrin family proteins occur on the plasma membrane (Ishikawa and Yoshida, 1985; Abdrakhamanova et al., 2003; Kosová et al., 2007). These changes were also confirmed by semi-proteomic analyses (Kawamura and Uemura, 2003). However, these studies were focused on specific proteins or at most on proteins that were identified on two-dimensional electrophoresis (2-DE) gels as cold-responsive proteins and not performed in a large scale to obtain comprehensive data sets of the candidate proteins in the plasma membrane. Proteomics of the plasma membrane with a help of rapid advance of analytical techniques using mass spectrometry will provide us information that suggests us a number of associations of the plasma membrane proteins and cold acclimation mechanism in a relatively short period. Thus, elucidation of proteome profiles of the plasma membrane when exposed to low temperature in combination with genetic and physiological studies will be valuable for practical improvement of plant cold tolerance.

## RESPONSES OF PLASMA MEMBRANE PROTEOME TO ABIOTIC STRESSES

In this section, we review studies of proteomic changes of the plasma membrane in association with abiotic stresses. Abiotic stresses (e.g., drought, salt, flooding, light, nutrition, physical pressure, and extreme temperatures) generally restrict plant distribution, growth and reproduction, and sometimes result in severe damage to plants. Plasma membrane proteomics have revealed the possibility that plants have adaptation systems against abiotic stress factors in association with plasma membrane functions. For example, the relationship between plasma membrane and salt stress has been extensively investigated. Nohzadeh et al. (2007) and Cheng et al. (2009) performed comprehensive proteome analysis of rice root plasma membranes based on 2-DE mapping. Nohzadeh et al. (2007) identified eight proteins that responded to salt stress. These proteins included 14-3-3 proteins, which are well known as proton-ATPase regulators (Babakov et al., 2000) and may be involved in pH regulation in cells under salt stress conditions. Cheng et al. (2009) also identified 18 salt-responsive proteins. Proteomic analyses combined with immuno-histochemical analysis of the proteins revealed that a leucine-rich-repeat type receptor-like protein kinase, OsRPK1, which responded to salt stress, accumulated in the cortex plasma membrane under salt stress conditions. Nouri and Komatsu (2010) and Komatsu et al. (2009) analyzed osmotic and flooding stress responses, respectively, of the soybean plasma membrane proteome. They identified dozens of stress-responsive plasma membrane proteins using 2-DE- and nano-LC-MS/MS based proteomics approaches. These results suggest that the plasma membrane has functions that are important for adaptation to osmotic and flooding stresses via ion homeostasis and antioxidative mechanisms.

## PLASMA MEMBRANE PROTEOMIC APPLICATIONS FOR INCREASING COLD TOLERANCE

**FIGURE 1:**
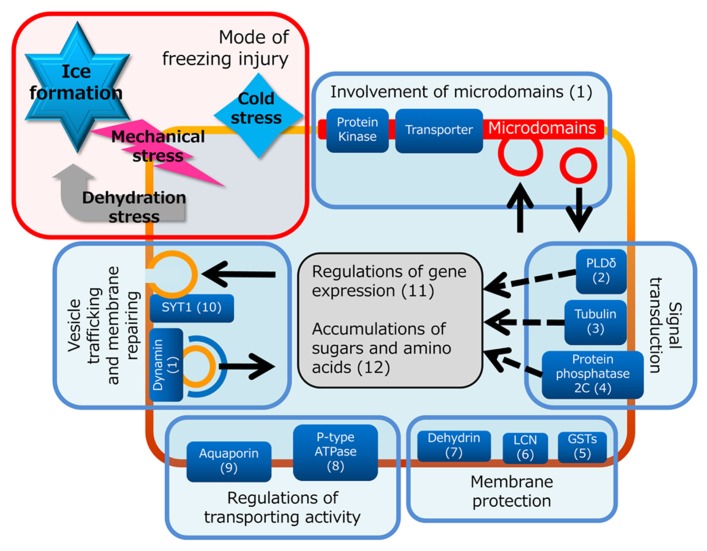
**Representative functions of the plasma membrane during cold acclimation suggested by previous proteomics studies.** Freezing temperature causes different types of injury including membrane destabilization and protein inactivation resulted from two primary stresses, dehydration stress and mechanical stress, imposed by ice crystals formed in extracellular space. Plant cells can respond to these stresses through signal transduction and gene regulation pathway, and counter these stresses with cold-evoked membrane protection system, optimization of transporting activity and adjustment of vesicle trafficking and membrane patch-up system. Each mechanism related to response to freezing has possibilities to be mediated by proteins and/or lipid environments in microdomains that consist of specific proteins and lipids. Detailed information of each part is referred to following papers: (1) Minami et al., 2009; (2) Li et al., 2004; (3) Abdrakhamanova et al., 2003; (4) Tähtiharju and Palva, 2001; (5) Seppänena et al., 2000; (6) Uemura et al., 2006; (7) Danyluk et al., 1998; (8) Ishikawa and Yoshida, 1985; (9) Peng et al., 2008; (10) Yamazaki et al., 2008; (11) Chinnusamy et al., 2007; (12) Koster and Lynch, 1992.

Temperature is one of the major regulatory factors of crop production. In particular, freezing temperatures are accompanied by a state change from water to ice in plants, which has dramatic effects on cellular metabolism. When plants are placed under low temperature conditions, the plasma membrane will also fall into functional decline (i.e., decline of membrane fluidity, transport and metabolic activity and ion homeostasis maintenance, disturbance of signaling processes, and freeze-induced fusion with other intracellular membranes because of physical pressure by extracellular ice formation). Plant plasma membrane, however, has the ability to adapt to freezing temperatures through cold acclimation processes and the plasma membrane proteome is thought to be tightly associated with determination of plant freezing tolerance (**Figure 1**). Compositional changes of plasma membrane proteins and lipids have been well described (Uemura and Yoshida, 1984; Uemura and Steponkus, 1994; Webb et al., 1994; Uemura et al., 1995). On the protein side, Kawamura and Uemura (2003) first applied mass spectrometric technology to elucidate the relationship between plasma membrane proteins and cold acclimation and successfully identified 38 proteins the levels of which changed during 3 days of cold acclimation treatment in *Arabidopsis*. These cold-responsive plasma membrane proteins include early responsive to dehydration proteins (ERD10 and ERD14), which are members of the dehydrins and may protect proteins and membranes against freeze-induced dehydration (Uemura et al., 2006; Kosová et al., 2007). In addition, a novel cold acclimation-induced protein, plant synaptotagmin 1 (SYT1), was identified. In animal cells, an isoform of the synaptotagmin family is known to be associated with membrane resealing (Reddy et al., 2001). Thus, they hypothesized that SYT1 is involved in repairing the plasma membrane when it is disrupted during the freeze-thaw cycle. Yamazaki et al. (2008) later confirmed that synaptotagmin-associated membrane resealing is eventually involved in plant freezing tolerance mechanism. Li et al. (2012) recently applied shotgun proteomics technology to show proteome profiles of the plasma membrane during cold acclimation or abscisic acid (ABA) treatment in *Arabidopsis *suspension cultured cells. ABA is well known to play a key role in plant cold acclimation (Chen and Gusta, 1983; Bakht et al., 2006). Their data suggested that a subset of cold acclimation-induced changes of plasma membrane proteins were mimicked by ABA treatment, but another set of proteins was characteristically changed only by cold acclimation treatment and not by ABA.

Furthermore, Minami et al. (2009) performed proteome profiling to characterize alterations of the *Arabidopsis* microdomain during cold acclimation. The microdomain is a lateral membrane subdomain composed of specific lipids and proteins in the plasma membrane (Simons and Ikonen, 1997). Microdomain proteomics offered new insight into changes and functions of the plasma membrane proteome during cold acclimation. Many functional proteins that are associated with cold acclimation, such as P-type ATPases, aquaporins, and tubulins (Ishikawa and Yoshida, 1985; Abdrakhamanova et al., 2003; Peng et al., 2008), accumulated in microdomain fractions. Minami et al. (2009) found that clathrins and dynamin-related proteins, which are associated with the clathrin-dependent endocytosis pathway, increased in the microdomain during cold acclimation. They predicted that functional changes of endocytosis activity during cold acclimation are regulated by microdomain-enriched clathrins and dynamin-related proteins. Recently, we reported plasma membrane microdomain proteomes isolated from oat and rye (Takahashi et al., 2012). Although oat and rye are monocotyledonous plants and *Arabidopsis *is a dicotyledonous plant, the microdomain proteome profiles of these three species were quite similar (Borner et al., 2005; Minami et al., 2009; Takahashi et al., 2012). Interestingly, oat and rye are phylogenetically related but have quite different freezing tolerance after cold acclimation (Webb et al., 1994). Now, we are conducting analysis to determine the relationship between alterations of the microdomain proteome during cold acclimation and freezing tolerance mechanism. Because monocotyledonous plants contain a number of important crops, such as rice and wheat as well as oat and rye, plasma membrane proteomics in monocotyledonous plants is important for practical improvement of crop freezing tolerance. Along with this line of research, we have just initiated a new project using shotgun proteomics to analyze the plasma membrane proteome during cold acclimation using a novel model monocotyledonous plant, *Brachypodium distachyon* (Draper et al., 2001; The International Brachypodium Initiative, 2010). Information from plasma membrane proteomes during cold acclimation in *B. distachyon* will contribute to the improvement of freezing tolerance in agriculturally important crops and increase the cultivated land available for these crops.

## SIGNIFICANCE OF THE PLASMA MEMBRANE PROTEOMICS IN APPLICATION TO BREEDING COLD-TOLERANT CROPS

As described above, the plasma membrane plays significant roles in signal perception and cellular homeostasis, and plasma membrane proteins are the most important factors in determining the environmental stress tolerance of plants. Thus, plasma membrane proteomics helps to understand how plants adapt to stressful conditions and to consider how we improve crop production in severe environments. In terms of plant freezing tolerance, expression of several plasma membrane proteins has been genetically modified, which resulted in an increase in freezing tolerance. For example, phospholipase Dδ (PLDδ), a plasma membrane-associated protein which hydrolyzes membrane phospholipids and generates phosphatidic acid (PA), has been confirmed to increase in its amount during cold acclimation by a proteomic approach using *Arabidopsis* (Kawamura and Uemura, 2003). Knock-out of *PLDδ* gene resulted in decreased freezing tolerance, and further, overexpression resulted in increased freezing tolerance when compared with that of wild-type plants (Li et al., 2004). After freezing, higher levels of PA were observed in *PLDδ*-overexpressed plants. Because PA has been thought to be a signal molecule (Bargmann and Munnik, 2006), PA-mediated cellular functions may have critical roles in enhancement of freezing tolerance. Lipocalin-like protein (temperature-induced lipocalin, TIL), another plasma membrane protein that responds to cold treatment (Kawamura and Uemura, 2003), has a positive effect in freezing tolerance. Overexpression of TIL resulted in higher survival rates at freezing temperature (Tominaga et al., 2006; Uemura et al., 2006). However, molecular mechanism of the effect of LCN on freezing tolerance is still to be determined. Information obtained from proteomic results has plowed ahead with findings of novel factors in association with cold tolerance and contributed to improvement of tolerance to low temperature under laboratory conditions. Now we are at the stage that the proteomic information and the knowledge of cold adaptation and freezing tolerance mechanisms can be combined and with helps of different approaches such as genetics and molecular biology, we can go further to organize collaborative research for application of the proteome information to conventional bleeding or genetic engineering of crops with candidate proteins revealed by proteomic approaches.

## FUTURE PERSPECTIVES

Much information about cold tolerance and the plasma membrane proteome is becoming available for the breeding of crops that are productive in cold conditions. Because the molecular mechanisms of freezing stress tolerance are known to share commonalities with tolerance to other abiotic stresses such as drought and salt stress (Mahajan and Tuteja, 2005), the knowledge of cold-responsive plasma membrane proteins may be useful for producing crops tolerant to multiple abiotic stresses.

## Conflict of Interest Statement

The authors declare that the research was conducted in the absence of any commercial or financial relationships that could be construed as a potential conflict of interest.
